# Chinese herbal medicine anticancer cocktail soup activates immune cells to kill colon cancer cells by regulating the gut microbiota-Th17 axis

**DOI:** 10.3389/fphar.2022.963638

**Published:** 2022-09-06

**Authors:** Xiaoli Nie, Zixiang Geng, Jianjun Liu, Li Qi, Zetian Wang, Te Liu, Jianguo Tang

**Affiliations:** ^1^ Department of Trauma-Emergency and Critical Care Medicine, Shanghai Fifth People’s Hospital, Fudan University, Shanghai, China; ^2^ Shanghai Geriatric Institute of Chinese Medicine, Shanghai University of Traditional Chinese Medicine, Shanghai, China; ^3^ Department of Acupuncture, Shanghai General Hospital,Shanghai Jiao Tong University, Shanghai, China; ^4^ Institute of Interdisciplinary Integrative Medicine Research, Shanghai University of Traditional Chinese Medicine, Shanghai, China

**Keywords:** colon cancer, chinese herbal medicine anticancer cocktail soup (CHMACS), gut microbiota, Th17, tumor immune evasion

## Abstract

Chinese herbal medicines are effective for treating colon cancer (CC). CC development is reportedly associated with gut microbiota dysbiosis and immune function dysregulation. Herein we explored the therapeutic effects of a Chinese herbal medicine anticancer cocktail soup (CHMACS) on mice with CC and also explored its regulatory effects on gut microbiota. *In vivo* experiments indicated that CHMACS significantly inhibited the proliferation and tumorigenicity of CC cells. Further, CHMACS treatment decreased the proportion of CD8^+^ T, natural killer, and Th17 cells. HPLC/MS analysis showed that CHMACS comprised 227 active components. 16S rRNA sequencing revealed, for example, an increase in the relative abundance of *uncultured_bacterium_g_Turicibacter* and a decrease in that of *uncultured_bacterium_g_Coriobacteriaceae_UCG-002* in gut microbiota of CHMACS-treated mice. Microbial diversity cluster analysis revealed that *Verrucomicrobia*, *Spirochaetes*, *Fusobacteria*, *Patescibacteria*, and *Firmicutes* contributed the most to fecal microbial diversity. Kyoto Encyclopedia of Genes and Genomes metabolic pathway analysis and clusters of orthologous groups of protein annotation indicated that CHMACS treatment induced amino acid metabolism and suppressed carbohydrate metabolism. Moreover, we found a strong association between changes in metabolites and immune cell maturation and activation. To summarize, our findings suggest that CHMACS kills CC cells by regulating gut microbiota and activating immune cells.

## Introduction

Colon cancer (CC) is the third most common cancer across the world and the second leading cause of cancer deaths (Cunningham et al., 2010; Brenner et al., 2014; Pages et al., 2018; Song et al., 2020). Colon carcinogenesis is closely associated with diet, lifestyle habits, and chronic gut inflammation. Colonoscopy has been proven to be effective for CC detection; however, considering the lack of evident signs and symptoms during the early stage as well as limitations associated with early diagnosis, most patients are diagnosed at the late stage of the disease (Cunningham et al., 2010; Brenner et al., 2014; Pages et al., 2018; Song et al., 2020). Prognosis remains poor and the 5-years survival rate is only 13.1% (Cunningham et al., 2010; Brenner et al., 2014; Pages et al., 2018; Song et al., 2020). Therefore, it is imperative to elucidate the molecular mechanisms underlying CC progression and discover new biomarkers for early diagnosis so as to identify potential targets for CC treatment.

A strong association reportedly exists between gut microbiota composition and tumor microenvironment (Guarner and Malagelada, 2003; O’Keefe, 2016; Garrett, 2019; Jia et al., 2021; Peuker et al., 2022). In the early stages of CC, gut microbiota influence tumor initiation by interfering with DNA repair mechanisms and enhancing Wnt signaling. For example, enterotoxigenic *Bacteroides fragilis*, which produces the *B. fragilis* toxin, is relatively more abundant in early CC than in advanced CC, whereas *Fusobacterium* is much more abundant in the gut flora of patients with advanced CC (Guarner and Malagelada, 2003; O’Keefe, 2016; Garrett, 2019; Jia et al., 2021; Peuker et al., 2022). Gut microbiota dysbiosis due to broad-spectrum antibiotics has been reported to result in the loss of antitumor activity of oxaliplatin, which evidently activates caspase-3 in ileal epithelial cells. Such findings suggest that anticancer agents exert their anticancer effects *via* gut microbiota. Gut microbiota are a precipitating factor to promote the maturity of CD4^+^ T-cells. Host-specific symbionts reportedly drive the development of Th17 and Treg cells and the activation of Th1, Tfh, and B cells. It also affects various cell types involved in CD8^+^ T-cells, B cells, and innate immune cells, including dendritic cells, macrophages, eosinophils, and mast cells (Guarner and Malagelada, 2003; O’Keefe, 2016; Garrett, 2019; Jia et al., 2021; Peuker et al., 2022). Studies have found that ileal immune characteristics are associated with the prognosis of proximal CC and sensitivity to standard chemotherapy. Furthermore, a study found that in patients with poor prognosis, the transcription of genes related to ileal immune response suppression, particularly that of Th17-related genes, was significantly upregulated.

On transplanting feces of patients with CC into aseptic mice, fecal microflora were found to weaken the chemotherapeutic efficacy of oxaliplatin (Guarner and Malagelada, 2003; O’Keefe, 2016; Garrett, 2019; Roberti et al., 2020; Jia et al., 2021; Peuker et al., 2022). In addition, oxaliplatin-treated ileal epithelial cells from sterile mice are reportedly unable to induce a protective immune response to CC; however, specific bacteria isolated from ileal microflora of patients with CC could restore this response (Guarner and Malagelada, 2003; O’Keefe, 2016; Garrett, 2019; Roberti et al., 2020; Jia et al., 2021; Peuker et al., 2022). Simultaneously, some studies have found that the inhibitory effects on CC can be considerably improved by introducing immunogenic bacteria into mice treated with oxaliplatin combined with PD-1 blockers (Guarner and Malagelada, 2003; O’Keefe, 2016; Garrett, 2019; Roberti et al., 2020; Jia et al., 2021; Peuker et al., 2022). These results validate that gut microbiota are closely related to CC occurrence and development and that the optimal activity of chemotherapeutic agents and immune checkpoint blockers depends on the integrity of gut microbiota.

Chinese herbal medicines (CHMs) play a key role in CC treatment (McCulloch et al., 2011; Su et al., 2014; Lin et al., 2017; Xiang et al., 2019; Wang et al., 2020). The guiding philosophy of CHM to treat tumors is to give equal attention to enhancing immunity and antitumor activity. Many CHMs have been proven to have both antitumor activity and immunity-enhancing effects (Luo et al., 2019; Peng et al., 2019; Chen et al., 2020; Wang et al., 2020). Honokiol, cucurbitacin, evodiamine, quercetin, and α-mangostin, i.e., the anticancer components of CHMs, have been found to substantially inhibit the activity of CC cells (Luo et al., 2019; Peng et al., 2019; Chen et al., 2020; Wang et al., 2020). Honokiol, a bisphenol compound isolated from various parts of *Magnolia*, promotes CC cell apoptosis by inhibiting YAP1 phosphorylation and Notch signal transduction (Luo et al., 2019; Peng et al., 2019; Chen et al., 2020; Wang et al., 2020). Cucurbitacin B/I causes cell cycle arrest and apoptosis of CC stem cells by downregulating the Notch signaling pathway (Luo et al., 2019; Peng et al., 2019; Chen et al., 2020; Wang et al., 2020). Evodiamine, a derivative of the CHM *Evodia rutaecarpa*, reportedly inhibits Wnt/β-catenin and Notch signal transduction, and also induces apoptosis of CC stem cells (Luo et al., 2019; Peng et al., 2019; Chen et al., 2020; Wang et al., 2020). Quercetin, which is abundant in mulberries, when used in combination with chemotherapeutic agents has been shown to effectively inhibit the proliferation and “stemness” of CC stem cells (Luo et al., 2019; Peng et al., 2019; Chen et al., 2020; Wang et al., 2020).

Th17 cells exert biphasic regulatory effects on inflammation and tumors (Murphy et al., 1996; Harrington et al., 2005; Knochelmann et al., 2018). IL-1β and IL-6 or IL-1β, and IL-23 promote the activation of the transcription factor ROR-γt, resulting in differentiation of naïve T-cells into Th17 cells (Murphy et al., 1996; Harrington et al., 2005; Knochelmann et al., 2018). In addition, Th17 cells secrete IL-17 to promote the secretion of IL-8 by tissue-resident immune cells, recruit neutrophils, and protect against extracellular bacteria and parasites. IL-17 promotes IL-22 secretion by immune cells and plays a tissue-protective role (Murphy et al., 1996; Harrington et al., 2005; Knochelmann et al., 2018). The Th17-related cytokine IL-17 also mediates pro- and antitumor processes that influence the development and progression of various cancers, being associated with variable clinical outcomes (Marques et al., 2021). It has been widely reported that the gut microbiota–Th17 axis is closely related to the occurrence and development of immune system diseases as well as tumors (Li et al., 2016; Wilck et al., 2017; Brevi et al., 2020; Liu et al., 2020; Xing et al., 2021). For example, Li et al. (2016) found that treatment with probiotics inhibited hepatocellular carcinoma growth in mice; this effect involved the regulation of IL-17 secretion and a major reduction in the number of IL-17-producing Th17 cells. Xing et al. (2021) observed that *Odoribacter splanchnicus* was abundant in colitis and colorectal cancer (Tak1^ΔM/ΔM^) mice and sufficient to induce intestinal Th17 cell development; furthermore, they reported that altered microbiota of Tak1^∆M/∆M^ mice promoted the IL-1β and IL-6 signaling pathways, which are essential for the induction of protective intestinal Th17 cells and resistance (Li et al., 2016; Xing et al., 2021). Collectively, these data indicate that the gut microbiota–Th17 axis plays a crucial role in antitumor immunity.

CHMs, such as *Prunella vulgaris*, vine root, and snakeberry, play a vital role in CC treatment. In this study, representative CHMs possessing anticancer and immunity-enhancing effects were mixed in a fixed mass ratio to formulate a CHM anticancer cocktail soup (CHMACS). This soup, invented by Prof. Te Liu (Shanghai Geriatric Institute of Chinese Medicine, Shanghai University of Traditional Chinese Medicine), mainly comprised extracts of 14 Chinese medicinal herbs. Through drug intervention in tumor-bearing mice, mass spectrometry (MS), and high-throughput 16S rRNA sequencing of gut microflora, we comprehensively evaluated the biological mechanisms *via* which CHMACS inhibits the activity of CC cells.

## Materials and methods

### Chinese herbal medicine anticancer cocktail soup

Briefly (Zhang et al., 2019), CHMACS (obtained from Shanghai Geriatric Institute of Chinese Medicine, Shanghai University of Traditional Chinese Medicine) mainly consisted of extracts of the following Chinese medicinal herbs: Medlar (10 g), raspberry (10 g), *Epimedium* (10 g), *Psoralea* (10 g), *Duchesneau indica* (10 g), dodder (10 g), *Pinellia ternate* (10 g), *Radix puerariae* (10 g), *Radix scutellaria* (10 g), mulberry (10 g), *Prunella spica* (10 g), Rattan pear root (10 g), and *Rehmannia glutinosa* (10 g). All of them were decocted for 2 h, and the filtrates were mixed and condensed to 2 g crude drug/ml, followed by storage at 4°C.

### Cell culture

The murine CC cell line CT26WT was purchased from the Cell Bank/Stem Cell Bank of the China Center for Type Culture Collection, Chinese Academy of Sciences. Cells were cultured in RPMI-1640 (Gibco, Billings, MT, United States) supplemented with 10% fetal bovine serum (Gibco) and antibiotics (100 U/mL penicillin and 100 μg/ml streptomycin) at 37°C in a humidified atmosphere of 5% CO_2_. Cells were cultured under the same conditions until passage 2 before further experiments.

### Chinese herbal medicine anticancer cocktail soup treatment and *in vivo* xenografts

Briefly (Zhang et al., 2019), logarithmically grown CT26WT cells (1 × 10^8^) were inoculated into BALB/c mice lower right side of back. After tumor growth became evident, mice with the same tumor volume were randomly divided into two groups: CHMACS-treated and saline-treated (control). Each experimental group comprised six mice.

For the xenograft experiment, CHMACS (200 µl/day) was orally administered to BALB/c mice. CHMACS dosage (D) was calculated according to the Meeh–Rubner conversion formula between humans and mice: D_mouse_ = D_human_ × (K_mouse_/K_human_), wherein K represents the conversion factor (K_mouse_ = 1 and K_human_ = 0.11). The calculated *D* for mice (approximately 0.02 kg body weight) was 9.09 ml/kg body weight/day, which was approximately equal to 198 µl/day for each mouse.

After 60 days, mice were sacrificed and tumors were surgically excised. Tumors were weighed, and tumor volume (mm^3^) was calculated as follows: (*ab* ^2^)/2, wherein *a* represents the longest axis (mm) and *b* represents the shortest axis (mm). All animal experiments were performed in accordance with the guidelines of the NIH for the care and use of laboratory animals. The study protocol was approved by the Committee on the Use of Live Animals in Teaching and Research, Shanghai Geriatric Institute of Chinese Medicine, Shanghai, China (SHAGE20210981).

### Hematoxylin–eosin staining

Tissue samples were fixed in 4% paraformaldehyde, dehydrated, and embedded in paraffin. Thin 4 μm-thick slices were then excised using a paraffin slicer and placed on a glass slide. Subsequently, the slices were dewaxed by xylene, followed by ethanol gradient dehydration. They were then stained with hematoxylin and incubated at 25°C for 5 min, differentiated with 1% hydrochloric acid/ethanol for 30 s, immersed into light ammonia water for 1 min to cause the nucleus to turn blue, and finally rinsed with distilled water for 5 min. Eosin was then added and incubated at 25°C for 2 min, followed by rinsing with distilled water for 2 min. Next, ethanol gradient decolorization was performed, followed by xylene permeation for 2 min. Finally, the slides were sealed with neutral gum for microscopy.

### Immunofluorescence staining

Briefly, all fresh tissues were soaked at 25°C and fixed in 4% paraformaldehyde (Sigma-Aldrich, St. Louis, MO, United States) for 30 min. Subsequently, they were subjected to ethanol gradient dehydration, and then paraffin embedded, sliced (thickness, 6 μm), and dewaxed in xylene. Next, they were sealed at 37°C for 30 min with immunohistochemical blocking solution (Beyotime Biotechnology, Zhejiang, China). The blocking solution was then discarded, and the sections were washed thrice with immunohistochemical cleaning solution (Beyotime Biotechnology) at 25°C for 5 min. A primary antibody was subsequently added, followed by incubation at 37°C for 45 min; the sections were then again rinsed three times with the cleaning solution at 25°C for 5 min. Next, a secondary antibody was added, followed by incubation at 37°C for 45 min. Finally, after cleaning the sections thrice with the cleaning solution at 25°C for 5 min, immunofluorescence sealing solution (Sigma-Aldrich) was added.

### Western blotting

Briefly, protein samples from each group were separated by 12% SDS-PAGE denaturing gel electrophoresis, and proteins were then transferred to a PVDF membrane (Millipore, Billerica, MA, United States). After sealing and washing the membrane, primary antibodies were added and incubated for 45 min at 37°C. After thoroughly washing the membrane, secondary antibodies were added and incubated for 45 min at 37°C. The membranes were then washed four times with TBST at 25°C for 14 min each time. Finally, protein bands were visualized with ECL^®^ enhanced chemiluminescence reagents (ECL kit; Pierce Biotechnology, Waltham, MA, United States), and band density was assessed by densitometry.

### Flow cytometry

Mouse peripheral blood mononuclear cells from each group were suspended (1 × 10^6^ cells/ml) and stained with primary antibodies on ice in Dulbecco’s phosphate-buffered saline containing 10% bovine serum albumin. Staining with an isotype control antibody (mouse IgG1-FITC, mouse IgG1-PE; eBioscience™; Invitrogen, Waltham, MA, United States) was performed to correct for non-specific binding. Antibody staining was analyzed by fluorescence correlation microscopy using the FACS Aria system (Quanta SC; Beckman Coulter, Brea, CA, United States).

### High-performance liquid chromatography/MS

HPLC/MS was performed by Icast Testing Technology Service (Shanghai, China). Briefly, 0.2 g CHMACS freeze-dried powder was dissolved in 8 ml 50% methanol aqueous solution, extracted for 40 min by ultrasound at 45°C, and centrifuged at 13,000 rpm for 10 min. The supernatant thus obtained was filtered through a 0.22-μm microporous membrane. This sample (200 μl) was analyzed by HPLC/MS using, which was performed on a Shimadzu LC-30 A system with a C18 column (1.7 μm, 2.1 × 100 mm). The column temperature was 35°C, flow rate was 0.3 ml/min, and injection volume was 5 μl. Separation was achieved using the gradient elution procedure and mobile phase, which consisted of acetonitrile–0.2% formic acid aqueous solution. MS was performed on an AB Sciex Triple TOF™ 5600 mass spectrometer, which was equipped with an electrospray ionization source in the positive ion mode. The voltage of the ion source was 5500 V, temperature was 500°C, de-cluster voltage was 100 V, collision energy was 35 eV, and collision energy spread was 15 eV. The first-order mass spectrum parent ion scanning Fan Tian was 50:1000, and the IDA setting response Bo exceeded the six peaks of 100 cps for secondary MS scanning. The sub-ion scanning range was 50–1000, and the open-state back deduction.

### Gut microbiota analysis

As previously described (Lin et al., 2020; Lai et al., 2021), fresh fecal samples were collected during the last 5 days of the experiment to analyze gut microbiota. Bacterial genomic DNA was extracted from frozen samples stored at −80°C. The V3 and V4 regions of the 16S rRNA gene were amplified by PCR using specific primers (forward: 5′-ACT​CCT​ACG​GGA​GGC​AGC​A-3′; reverse: 5′-GGACTACHVGGGTWTCTAAT-3′). High-throughput pyrosequencing of PCR amplicons was performed on an Illumina sequencing platform at Biomarker Technologies (Beijing, China). Raw paired-end reads from the original DNA fragments were merged using FLASH32 and assigned to each sample, according to unique barcodes (Li et al., 2022). UCLUST in QIIME (v1.8.0) was used based on 97% sequence similarity. Tags were clustered into operational taxonomic units (OTUs). The α-diversity index was evaluated using Mothur v1.30. The diversity index was compared among samples by standardizing the number of sequences in each sample. OTU rank curves, rarefaction curves, and Shannon curves were constructed, and Shannon, Chao, Simpson, and abundance-based coverage estimator indices were calculated. For β-diversity analysis, heatmaps of RDA-identified key OTUs, principal co-ordinate analysis, non-metric multidimensional scaling, and unweighted pair group method with arithmetic mean were obtained using QIIME v1.8.0. The linear discriminant analysis (LDA)-effect size method was used for quantitative analysis of biomarkers. The LDA-effect size method (LDA score >4.0), non-parametric factorial Kruskal–Wallis sum-rank test, and unpaired Wilcoxon rank-sum test were applied to identify taxa with significantly different abundance.

### Statistical analysis

Each experiment was performed at least thrice; values represent mean ± standard error, unless stated otherwise. Differences were evaluated using Student’s *t*-tests (*n* = 4). *p* < 0.05 indicated statistical significance.

## Results

### Chinese herbal medicine anticancer cocktail soup inhibited the activity of colon cancer cells *in vivo*

**FIGURE 1:**
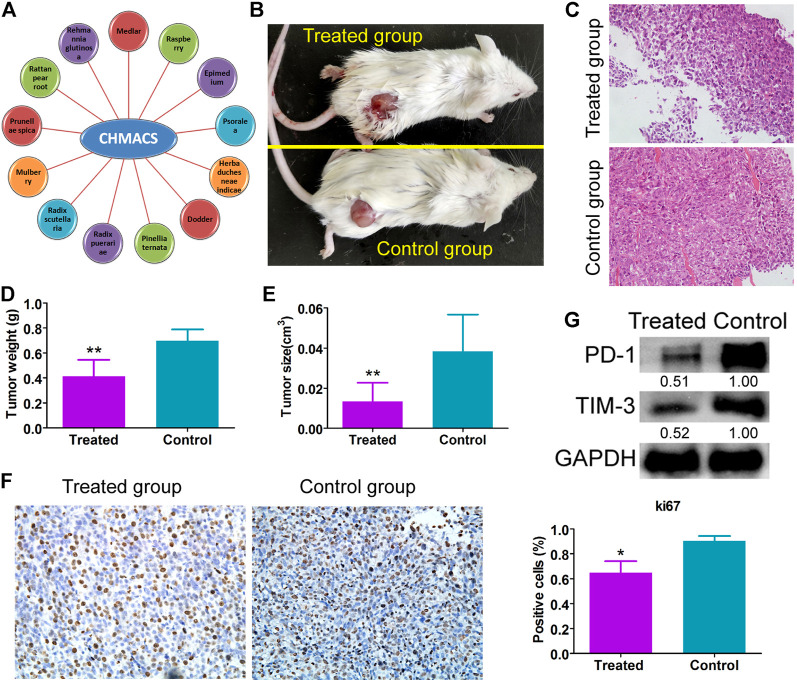
CHMACS inhibits the activity of CC cells *in vivo* **(A)** Composition of CHMACS. **(B)** Tumor morphology. **(C)** Hematoxylin*–*eosin staining of tumor tissues; **p < 0.01 vs. control; *n* = 6; *t*-test. **(D)** Tumor weight and **(E)** volume; **p < 0.01 vs. control; *n* = 6; *t*-test. **(F)** Immunohistochemical staining to assess proportion of cells that were positive for Ki67; *p < 0.05 vs. control; *n* = 6; *t*-test. **(G)** Western blotting data.

As explained earlier, CHMACS comprises CHMs with anticancer and immunity-enhancing effects (Figure 1A). Herein analyses of tumor-bearing mice revealed that tumor weight and volume were lower in the CHMACS-treated group as compared to those in the saline-treated (i.e., control) group (Figures 1B–E). Further, on hematoxylin*–*eosin staining of tumor tissues belonging to the CHMACS-treated and control groups, the results were consistent with the pathological characteristics of CC; however, the CHMACS-treated group exhibited significantly lower malignant degree than the control group (Figure 1C). Concurrently, immunohistochemical findings showed that relative to the control group, the proportion of cells that were positive for the nuclear proliferation factor Ki67 in the CHMACS-treated group was significantly lower (Figure 1F). Western blotting demonstrated that PD-1 and TIM-3 protein levels in peripheral blood mononuclear cells in the CHMACS-treated group were significantly lower than those in the control group (Figure 1G), suggesting that the activity of immune cells in the CHMACS-treated group was significantly enhanced. Collectively, these results suggested that CHMACS significantly inhibited not only the growth of CC cells but also their ability to evade the immune response in tumor-bearing mice.

### Chinese herbal medicine anticancer cocktail soup stimulated the activity of immune cells

**FIGURE 2:**
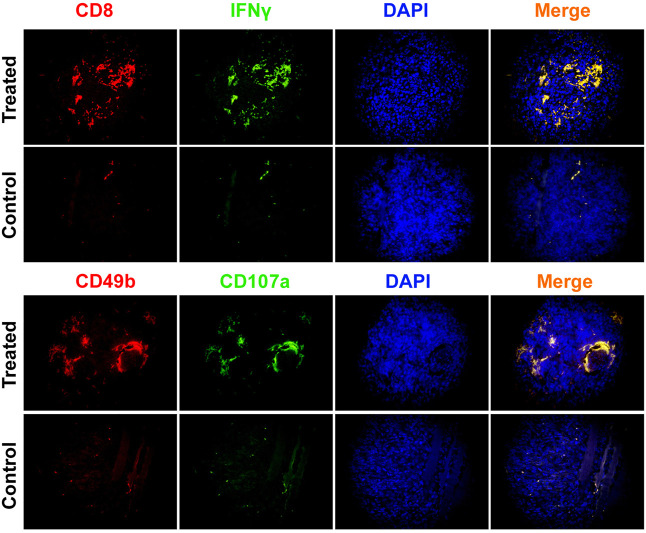
Immunofluorescence staining. In comparison to the control group, proportion of CD8+/IFNγ+ cells, and CD49b+/CD107a+ cells in tumor tissues belonging to the CHMACS-treated group was significantly higher.

**FIGURE 3:**
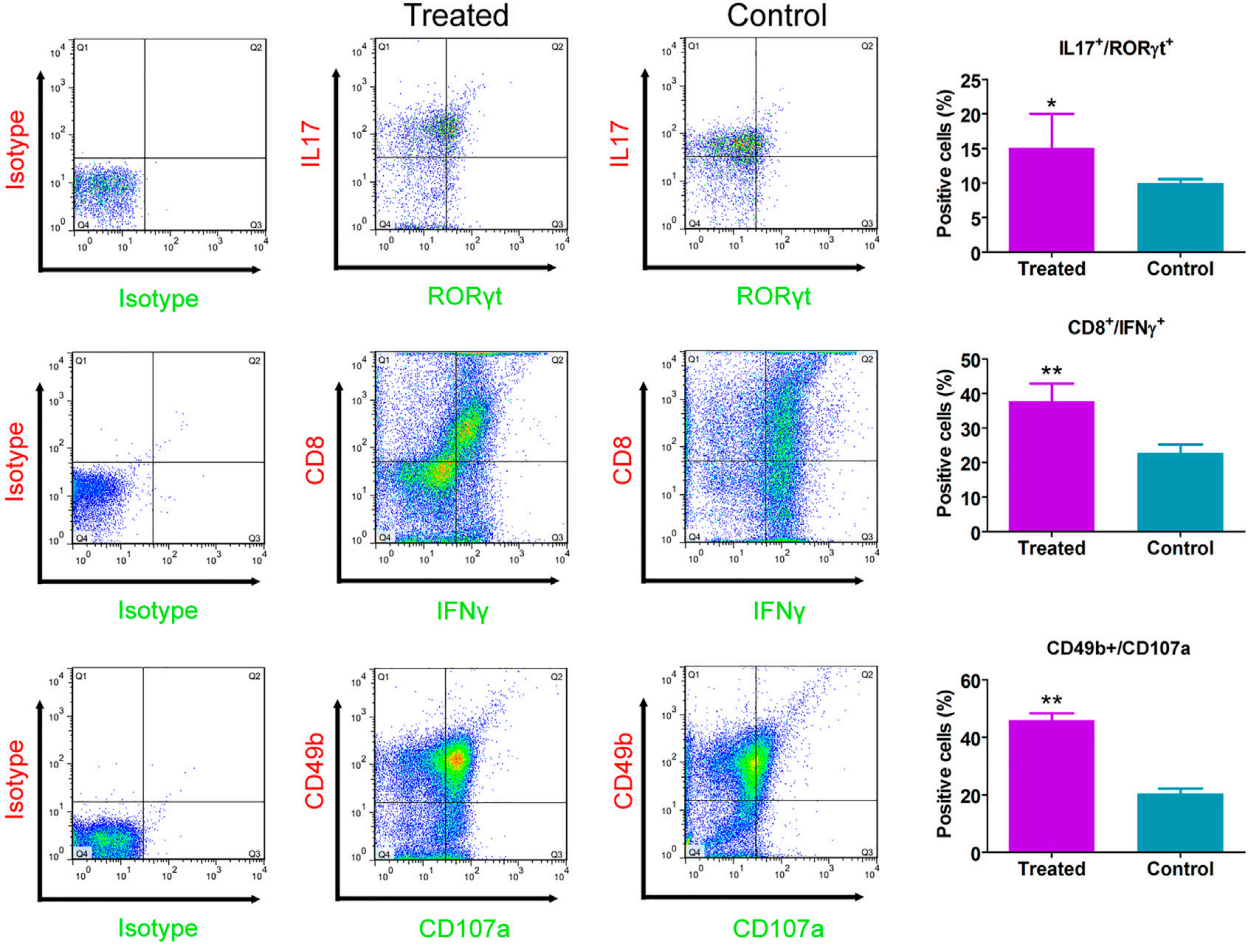
Flow cytometry analysis. Relative to the control group, proportion of IL17^+^/RORγt^+^, CD8^+^/IFNγ^+^, and CD49b^+^/CD107a^+^ cells in the peripheral blood of mice in the CHMACS-treated group was significantly higher. **p < 0.01 vs. control; *p < 0.05 vs. control; *n* = 6; *t*-test.

Immunofluorescence staining results showed that relative to the control group, the number of CD8^+^/IFNγ^+^, and CD49b^+^/CD107a^+^ cells in the CHMACS-treated group was significantly higher (Figure 2). Moreover, flow cytometry data showed that the proportion of IL17^+^/RORγt^+^, CD8^+^/IFNγ^+^, and CD49b^+^/CD107a^+^ cells in peripheral blood samples obtained from the CHMACS-treated group was significantly higher than in those from the control group (Figure 3). These results indicated that CHMACS significantly activated Th17, CD8, and natural killer cells in mice.

### Chinese herbal medicine anticancer cocktail soup comprised various anticancer and immunity-boosting active ingredients

**FIGURE 4:**
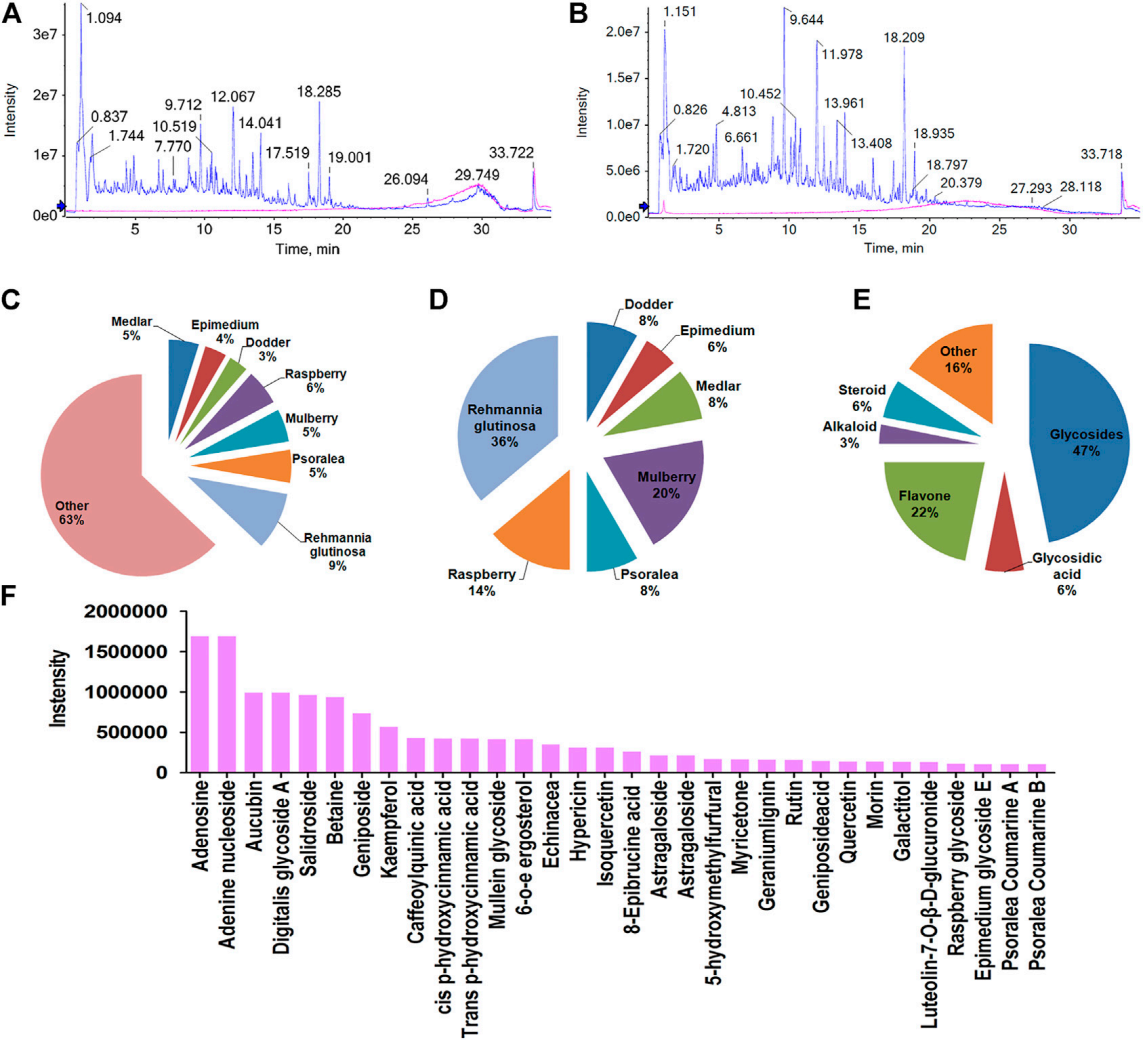
Chemical composition of CHMACS. HPLC/MS spectra of CHMACS in the **(A)** positive and **(B)** negative ion modes. **(C)** Distribution of chemical components of CHMACS and **(D)** of those with intensity >100,000. These active components were mainly derived from *R. glutinosa*, mulberry, raspberry, *Psoralea*, and other CHMs. **(E)** Classification of the active components of CHMACS based on their chemical properties. **(F)** Chemical components of CHMACS according to their intensity.

CHMACS analyses under the positive and negative ion modes led to the identification of 227 active components (Figures 4A,B,F); of these, 74 showed an intensity of >100,000. According to the response intensity of MS, these included, for example, trehalose, adenosine, aucubin, rehmannioside A, salidroside, betaine, kaempferol, ω-hydroxyemodin, luteolin, acteoside, and hyperoside (Table 1). On classifying and analyzing all active components detected by MS, we found that they were mainly derived from *R. glutinosa*, raspberry, mulberry, *Psoralea*, and other CHMs (Figure 4C). Further, on classifying active components with an intensity of >100,000, it became evident that these ingredients were predominantly derived from *R. glutinosa*, mulberry, raspberry, and other CHMs (Figure 4D). As per their chemical properties, the active components mainly included glycosides, flavones, glycosidic acid, steroids, amongst others (Figures 4E, F).

**TABLE 1:** Detection results of active components of herb by mass spectrometry.

Drug	Name2	Formula	Adduct	Found Mass	Instensity
Raspberry	Adenosine	C_10_H_13_N_5_O_4_	+H	268.10359	1689588
Rehmannia glutinosa	Adenine nucleoside	C_10_H_13_N_5_O_4_	+H	268.10359	1689588
Rehmannia glutinosa	Aucubin	C_15_H_22_O_9_	−H	345.1189	992341
Rehmannia glutinosa	Digitalis glycoside A	C_15_H_22_O_9_	−H	345.1189	992341
Rehmannia glutinosa	Salidroside	C_14_H_20_O_7_	−H	299.11362	960607
Medlar	Betaine	C_5_H_11_NO_2_	+H	118.08673	935586
Rehmannia glutinosa	Geniposide	C_17_H_24_O_10_	−H	387.12917	735799
Dodder	Kaempferol	C_15_H_10_O_6_	+H	287.05475	567103
Mulberry	Caffeoylquinic acid	C_16_H_18_O_9_	−H	353.0873	429134
Medlar	*cis* p-hydroxycinnamic acid	C_9_H_8_O_3_	+H	165.05448	422945
Medlar	*Trans* p-hydroxycinnamic acid	C_9_H_8_O3	+H	165.05448	422945
Rehmannia glutinosa	Mullein glycoside	C_29_H_36_O_15_	−H	623.19688	414557
Rehmannia glutinosa	6-o-e ergosterol	C_29_H_36_O_15_	−H	623.19688	414557
Rehmannia glutinosa	Echinacea	C_35_H_46_O_20_	−H	785.25054	348399
Dodder	Hypericin	C_21_H_20_O_12_	−H	463.08694	310053
Raspberry	Hypericin	C_21_H_20_O_12_	−H	463.08694	310053
Mulberry	Isoquercetin	C_21_H_20_O_12_	−H	463.08694	310053
Rehmannia glutinosa	8-Epibrucine acid	C_16_H_24_O_10_	−H	375.12975	260018
Dodder	Astragaloside	C_21_H_20_O_11_	−H	447.09226	216533
Raspberry	Astragaloside	C_21_H_20_O_11_	−H	447.09226	216533
Mulberry	Astragaloside	C_21_H_20_O_11_	−H	447.09226	216533
Psoralea	Astragaloside	C_21_H_20_O_11_	−H	447.09226	216533
Rehmannia glutinosa	5-hydroxymethylfurfural	C_6_H_6_O_3_	+H	127.03929	167991
Mulberry	Myricetone	C_15_H_10_O_8_	+H	319.04463	165541
Rehmannia glutinosa	Geraniumlignin	C_16_H_12_O_6_	+H	301.07062	161340
Raspberry	Rutin	C_27_H_30_O_16_	−H	609.14536	159065
Mulberry	Rutin	C_27_H_30_O_16_	−H	609.14536	159065
Rehmannia glutinosa	Geniposideacid	C_16_H_22_O_10_	+H	375.12708	145237
Mulberry	Quercetin	C_15_H_10_O_7_	+H	303.0496	137230
Mulberry	Morin	C_15_H_10_O_7_	+H	303.0496	137230
Epimedium	Galactitol	C_6_H_14_O_6_	−H	181.07216	135399
Rehmannia glutinosa	Luteolin-7-O-β-D-glucuronide	C_21_H_18_O_12_	+H	463.0866	131141
Raspberry	Raspberry glycoside	C_30_H_44_O_5_	+H	485.32568	112788
Epimedium	Epimedium glycoside E	C_20_H_16_O_6_	−H	351.08761	106123
Psoralea	Psoralea Coumarine A	C_20_H_16_O_6_	−H	351.08761	106123
Psoralea	Psoralea Coumarine B	C_20_H_16_O_6_	−H	351.08761	106123

### Regulation of the distribution and metabolism of gut microflora by CHMACS in tumor-bearing mice

**FIGURE 5:**
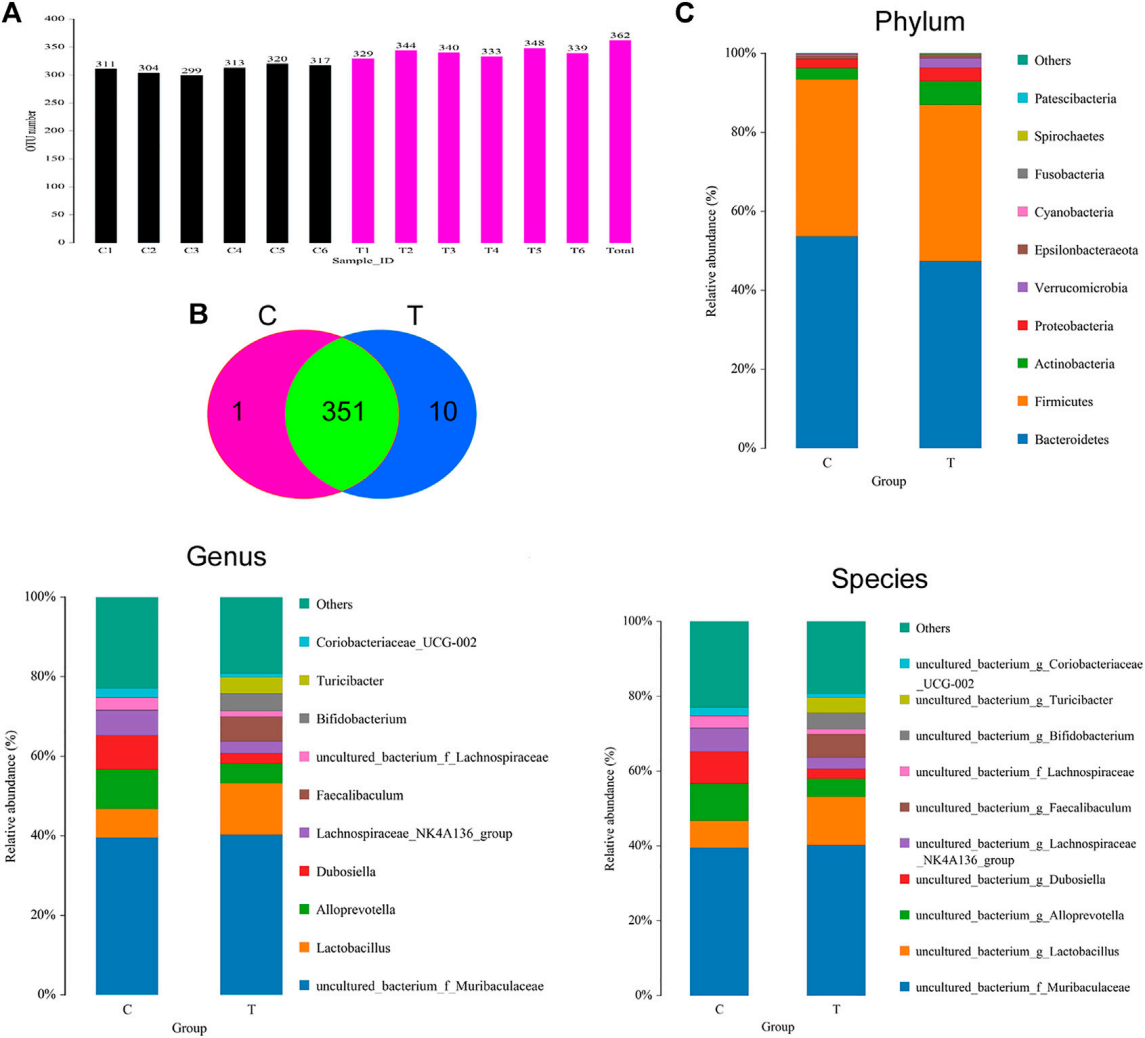
Analysis of OTUs **(A)** Number of OTUs.**(B)** Venn diagram; as evident, 351 OTUs were common between the groups. The CHMACS-treated group showed 10 unique OTUs, while the control group showed only one unique OTU. **(C)** Gut microbiota clustering and species distribution.

The feces of mice in the CHMACS-treated and control groups were collected, and the composition and distribution of gut microflora were evaluated by sequencing the V3 and V4 regions of the 16s rRNA gene. The SILVA database was used for analyses. Overall, 960,667 pairs of reads were obtained from 12 samples. A total of 950,076 clean tags were produced after double-ended read splicing and filtration, with at least 78,631 clean tags per sample and average of 79,173 clean tags. Based on 97% sequence similarity, UCLUST in QIIME (v1.8.0) was used for clustering tags into OTUs. The CHMACS-treated and control groups showed a significant difference in the number of OTUs (Figure 5A). In total, 351 OTUs were common between the groups; the CHMACS-treated group included 10 unique OTUs, whereas the control group included only one unique OTU (Figure 5B). On comparing the representative sequence of OTUs with the microbial reference database, each OTU could be divided into one species. Concomitantly, the community composition of each sample was determined. Phylum-level analysis indicated that the relative abundance of *Actinobacteria* and *Verrucomicrobia* was significantly higher and that of *Bacteroidetes* was significantly lower in the CHMACS-treated group than that in the control group. Genus-level analysis revealed that in comparison to the control group, the relative abundance of *Turicibacter*, *Faecalibaculum*, *Lactobacillus*, and *Bifidobacterium* was significantly higher in the CHMACS-treated group and that of *Lachnospiraceae_NK4A136_group*, *uncultured_bacterium_f_Lachnospiraceae*, *Dubosiella*, and *Alloprevotella* was significantly lower. Species-level analysis indicated that the relative abundance of *uncultured_bacterium_g_Turicibacter*, *uncultured_bacterium_g_Faecalibaculum*, and *uncultured_bacterium_g_Lactobacillus* was significantly higher in the CHMACS-treated group than that in the control group. In contrast, the relative abundance of *uncultured_bacterium_g_Coriobacteriaceae_UCG-002*, *uncultured_bacterium_g_Dubosiella*, *uncultured_bacterium_g_Lachnospiraceae_NK4A136_group*, and *uncultured_bacterium_f_Lachnospiraceae* was significantly lower in the CHMACS-treated group (Figures 5C–E).

**FIGURE 6:**
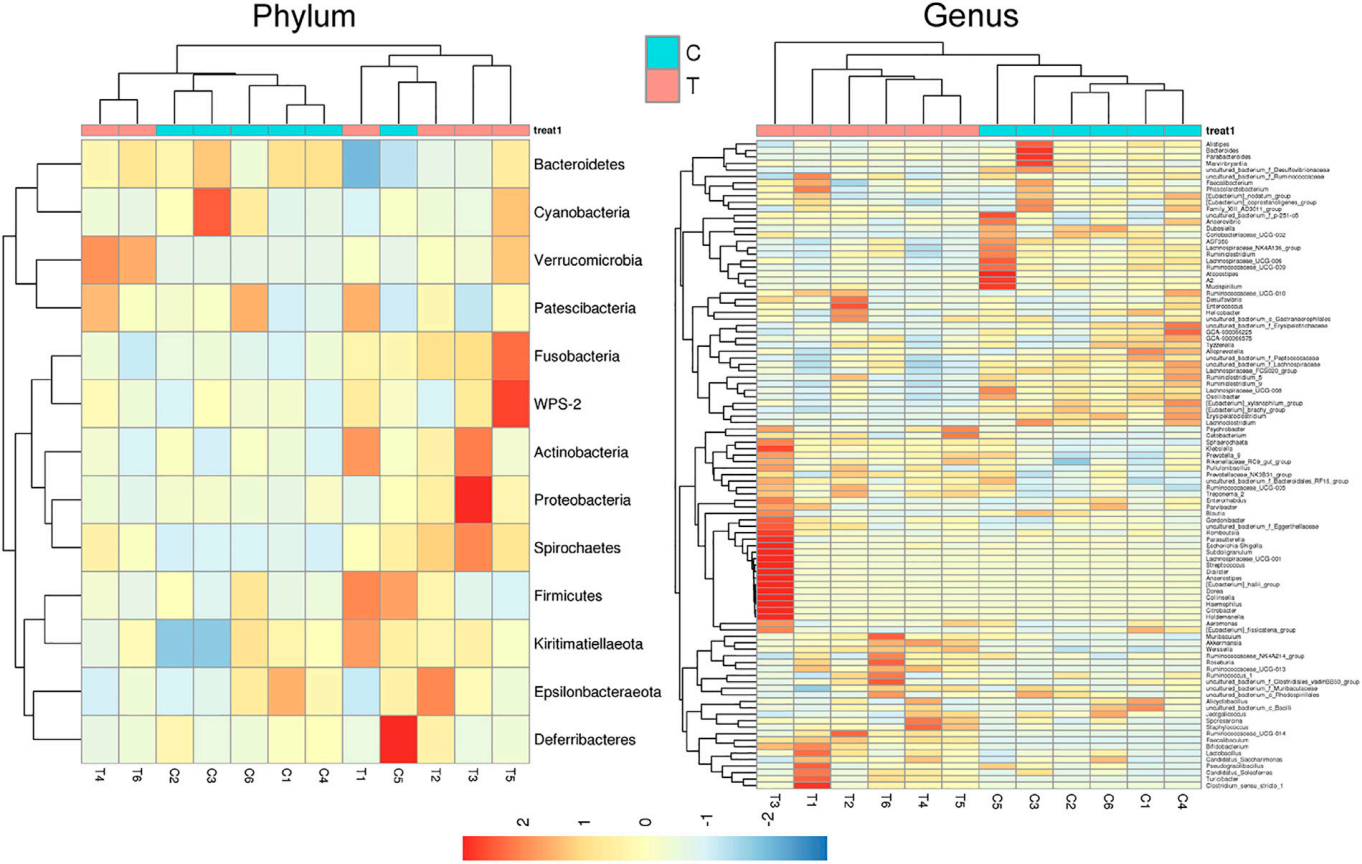
Microbial diversity clustering. **(A)** Phylum-level analysis; the relative abundance of *Actinobacteria* and *Verrucomicrobia* in the CHMACS-treated group was significantly higher than that in the control group. **(B)** Genus-level analysis; the relative abundance of *Turicibacter*, *Faecalibaculum*, *Lactobacillus*, and *Bifidobacterium* in the CHMACS-treated group was significantly higher than that in the control group.

**FIGURE 7:**
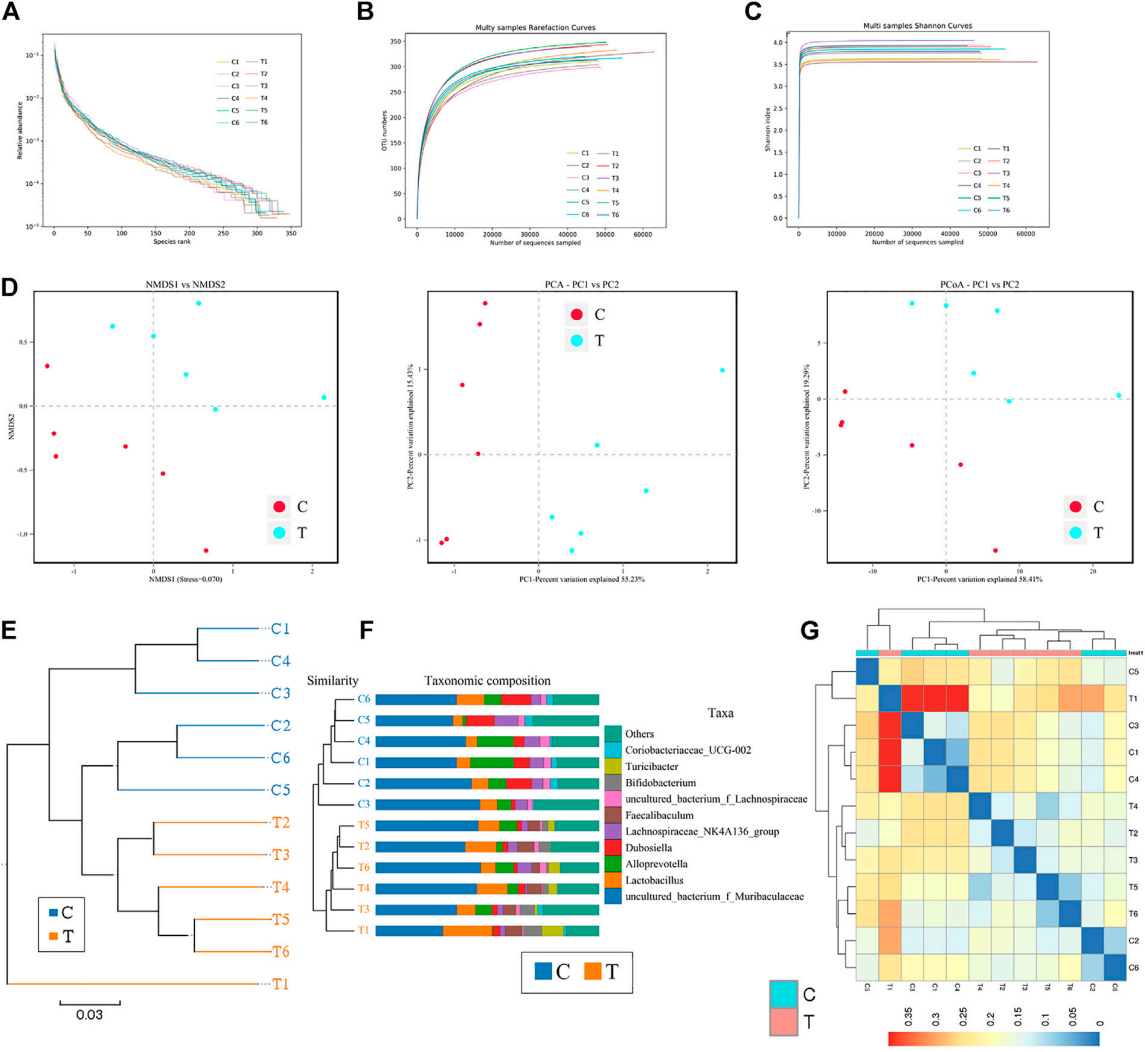
α- and β-diversity analysis **(A)** As evident, α-diversity analysis showed that the rank abundance curve was flat, indicative of a high degree of uniformity in species composition. **(B)** The rarefaction curve appeared smooth. **(C)** Shannon index curve. **(D)** Principal component analysis, principal co-ordinate analysis, and non-metric multidimensional scaling analysis. Microbial communities between the CHMACS-treated and control groups showed obvious differences. **(E)** Unweighted pair group method with arithmetic mean clustering. **(F)** Clustering and histogram combination. **(G)** Heatmap of sample abundance.

**FIGURE 8:**
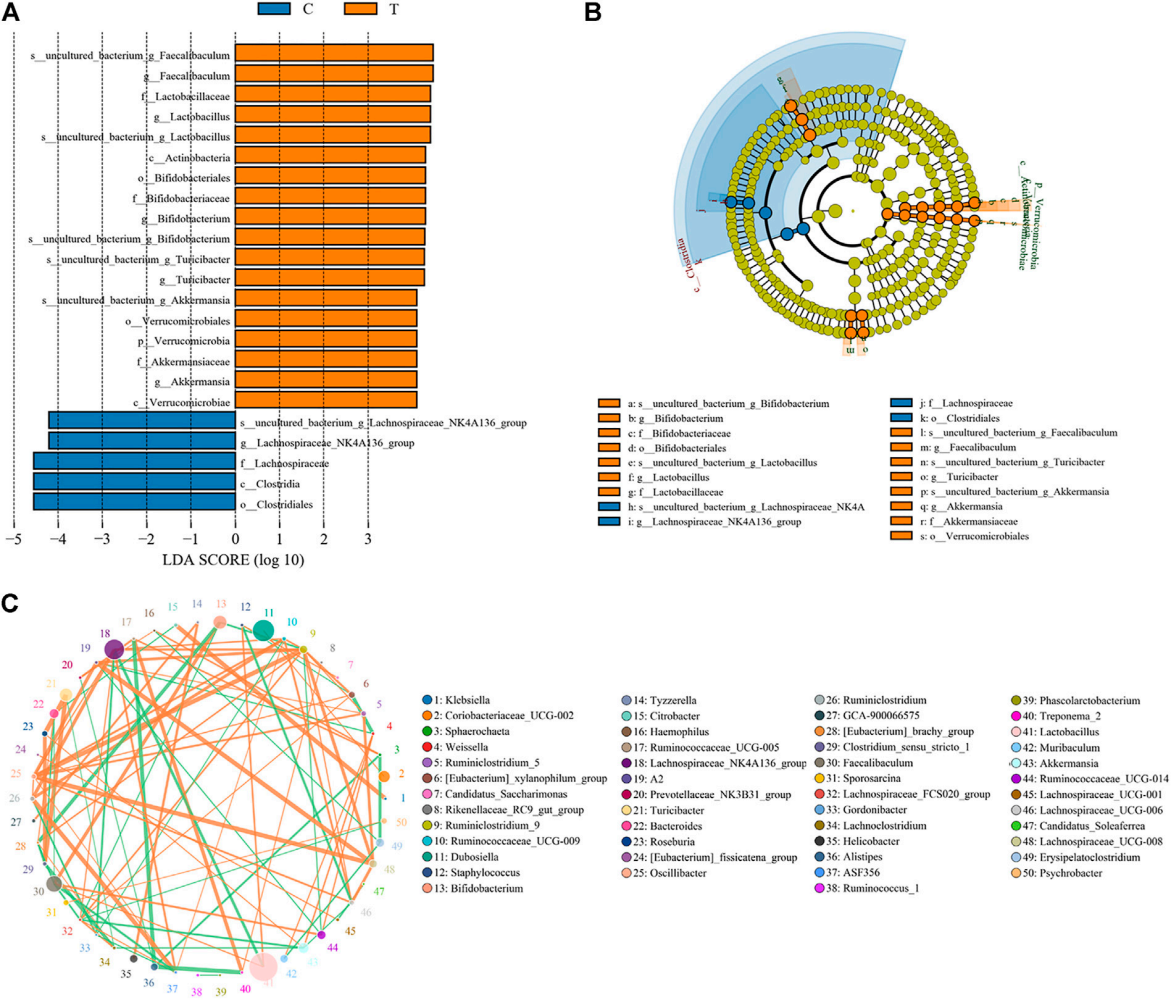
Significant difference analysis. **(A)** Value distribution histogram of line discriminant analysis effect size. **(B)** Species annotation was visualized using KRONA. **(C)** Species network at the genus level.

Microbial diversity cluster analyses demonstrated that in the CHMACS-treated group, microorganisms belonging to, for example, *Verrucomicrobia*, *Spirochaetes*, *Fusobacteria*, *Patescibacteria* (all increased), and *Firmicutes* (decreased), contributed the most to fecal microbial diversity (Figures 6A, B). In addition, α-diversity analysis showed that the rank abundance curve was flat, indicative of a high degree of uniformity in species composition (Figure 7A). The rarefaction curve was smooth, and the sample sequence was sufficient; hence, data analysis was feasible (Figure 7B). The Shannon index tended to be flat, indicating that the amount of sequencing data was sufficient and that the number of OTUs did not increase with the expansion of extracted data (Figure 7C). The β-diversity analysis primarily included principal co-ordinate analysis, principal component analysis, and non-metric multidimensional scaling, which revealed obvious differences in microbial communities between the CHMACS-treated and control groups; in other words, microorganisms in the two groups formed distinct communities (Figure 7D). Hierarchical cluster analysis of the unweighted pair group method with arithmetic mean showed a low level of homology of alveolar microbiota between the CHMACS-treated and control groups, and genetic background was distinct as well (Figures 7E–G). In addition, we applied the LDA-effect size method to identify high-dimensional biomarkers of intestinal microflora. LDA score >4.0 was indicative of a key biomarker. As evident from the results of cladogram analysis and LDA score distribution, in the CHMACS-treated group, the abundance of *g_Faecalibaculum*, *f_Lactobacillaceae*, *c_Actinobacteria*, *o_Bifidobacteriales*, *g_Turicibacter*, and *o_Verrucomicrobiales* was significantly higher than that in the control group, implying that these were the endemic microbial communities in the CHMACS-treated group (Figures 8A–C). In the control group, *g_Lachnospiraceae_NK4A136_group*, *f_Lachnospiraceae*, *c_Clostridia*, and *o_Clostridiales* represented the endemic microbial communities (Figures 8A–C).

### Chinese herbal medicine anticancer cocktail soup regulated the differential expression of functional genes and metabolic pathways of gut microflora

**FIGURE 9:**
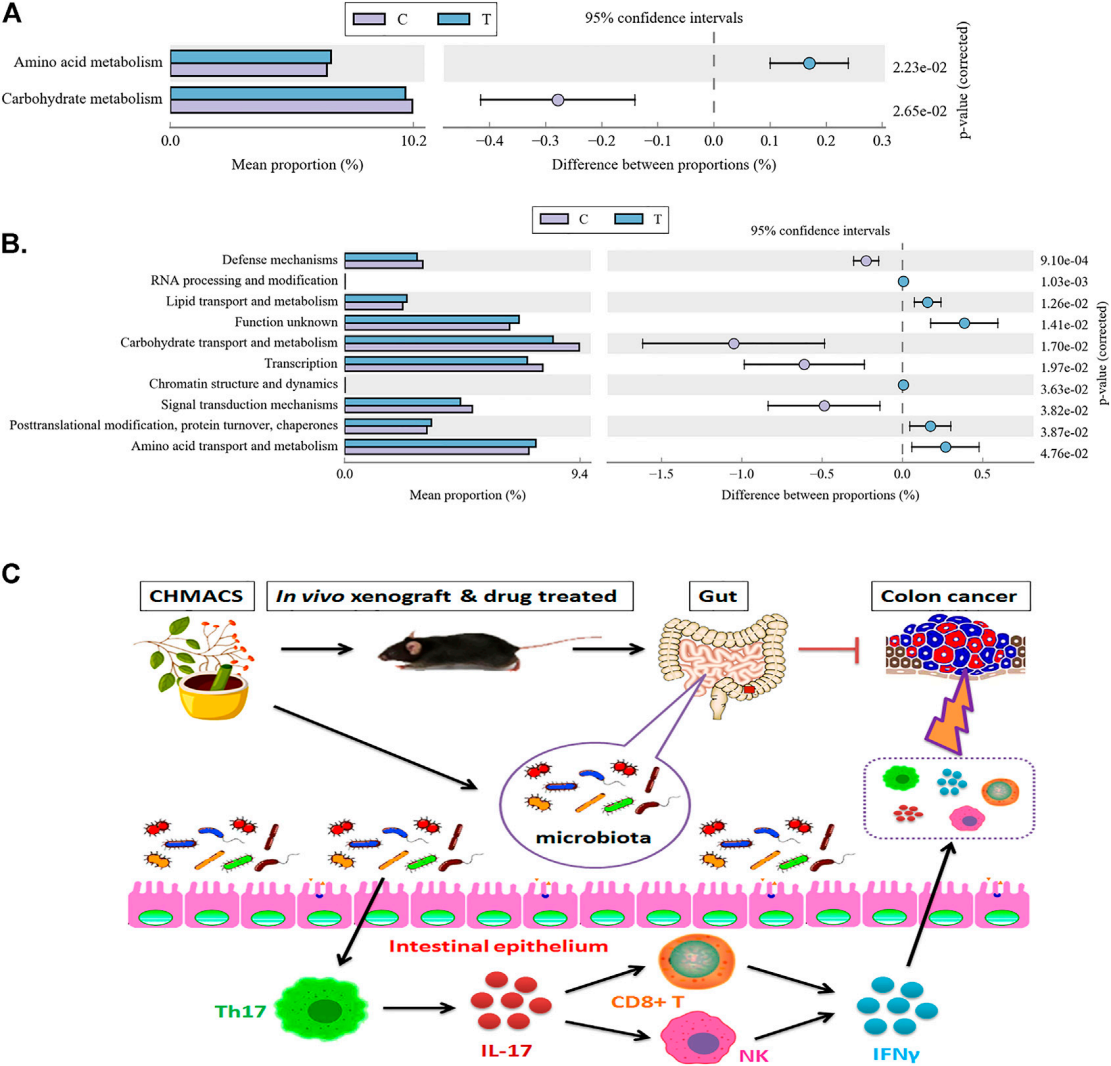
Metabolic signaling pathways and protein differences in gut microbiota. **(A)** Kyoto Encyclopedia of Genes and Genomes metabolic pathway analysis. **(B)** Analysis of clusters of orthologous groups of proteins to evaluate distribution and abundance of homologous protein clusters in gut microbiota. **(C)** CHMACS activates immune cells, which kill CC cells, by regulating the gut microbiota–Th17 axis.

Kyoto Encyclopedia of Genes and Genomes pathway analysis indicated changes in functional genes of microbial communities as a result of differential metabolic pathway activation. In comparison to the control group, amino acid metabolism was induced and carbohydrate metabolism was suppressed in the CHMACS-treated group (Figure 9A).

Analyzing clusters of orthologous groups of proteins can reveal the distribution and abundance of homologous protein clusters. Herein we found that in comparison to the control group, the abundance of nucleotide transport and metabolism (related to metabolism), post-translational modification, protein turnover, and chaperone (related to cellular processes and signaling) proteins was significantly higher in the CHMACS-treated group and that of carbohydrate transport and metabolism (related to metabolism), transcription (related to information storage and processing), and signal transduction mechanism (related to cellular processes and signaling) proteins was significantly lower (Figure 9B).

## Discussion

CHMs, either individual or in combination, have been proven to exert significant antitumor effects; moreover, they evidently not only inhibit the self-proliferation of tumor cells but also kill tumor cells by improving immunity (McCulloch et al., 2011; Su et al., 2014; Lin et al., 2017; Xiang et al., 2019; Wang et al., 2020). These effects are attributable to specific active components or active ingredients in CHMs (McCulloch et al., 2011; Su et al., 2014; Lin et al., 2017; Xiang et al., 2019; Wang et al., 2020). In this study, CHMs with both immunomodulatory effects and antitumor activity were used to derive CHMACS. HPLC/MS revealed the presence of several chemically active components in CHMACS, most with the ability to enhance immunity. *In vivo* experiment findings corroborated that CHMACS considerably inhibited tumor formation and tumor cell proliferation. We hypothesized that CHMACS inhibits tumor formation by stimulating immune activity in tumor-bearing mice. Subsequently, flow cytometry data led to the identification of several common types of immune cells that are directly related to tumor killing. Our data also confirmed our conjecture that the proportion of activated CD8^+^ T, natural killer (high expression of IFNγ or CD107a), and Th17 cells in tumor tissues and peripheral blood of CHMACS-treated mice was markedly higher. These results clearly indicated that relative to control mice, tumor-bearing mice treated with CHMACS showed significantly higher immune activity levels. Further, we assessed active components of CHMACS and their antitumor effects. Based on our literature search, some of the aforementioned active components were found to have major antitumor effects. For example, Li et al. (2022) recently reported that the gut of kaempferol-treated mice was predominantly colonized by short-chain fatty acid- and lactic acid-producing bacteria; in addition, kaempferol was found to effectively attenuate colorectal tumor burden in Apc^Min/+^ mice by modulating bile acid signaling and gut microbiota homeostasis. Shao et al. (2022) found that aucubin alleviated breast cancer-induced organ inflammatory damage without any visible side-effects; besides, aucubin increased the expression of colonic tight junction protein and adjusted the gut microbiome to normal levels. Similarly, Zhang et al. (2022) showed that acteoside improved cancer-related fatigue by inducing skeletal muscle mitophagy *via* PHD2 suppression to remove dysfunctional mitochondria, indicating the potential of acteoside for clinical applications. These results confirm the anticancer effects of active components in CHMACS. Further studies are warranted to elucidate the molecular mechanisms *via* which immune cells are activated to exert antitumor effects.

CHMACS enters the blood stream through intestinal cells when orally administered, implying that gut microbiota play a crucial role in CHMACS processing and absorption. Therefore, in this study, we comprehensively investigated the structure and composition of gut microbiota in tumor-bearing mice before and after administering CHMACS; moreover, we elucidated the effects of CHMACS on the immune system. In humans, gut microbiota are mainly dominated by thick-walled phylum and also *Bacteroides*, *Actinomycetes*, and *Proteus* phyla (Song et al., 2020). Gut microbiota metabolize indigestible components in foods, synthesize vitamins and other nutrients, detoxify metabolic enzymes, regulate immune response, provide signals for epithelial cell renewal and maintenance of mucosal integrity, and secrete antibacterial substances. Alterations in the abundance and composition of healthy gut microbiota leads to intestinal ecological imbalance, gradually resulting in chronic inflammation and production of carcinogenic metabolites, which consequently causes tumor formation. Gut microbiota in patients with CC can be divided into three distinct modes: the first mode involves continuous increase in the abundance of some pro-inflammatory bacteria (e.g., *Fusobacterium nucleatum* and *Peptostreptococcus stomatis*) from stage 0 to the more advanced stage; in the second mode, the abundance of *Atopobium parvulum* and *Actinomyces odontolyticus* increases in polyposis adenoma and/or stage 0 but remains unchanged during later stages; finally, in the third mode, the abundance of anti-inflammatory microorganisms or some probiotics, such as butyrate producers (e.g., *Lachnospira multipara* and *Eubacterium eligens*) decreases as the tumor progresses (Jia et al., 2021). In this study, our sequencing data revealed that the relative abundance of *uncultured_bacterium_g_Turicibacter*, *uncultured_bacterium_g_Faecalibaculum*, and *uncultured_bacterium_g_Lactobacillus* in gut microbiota of tumor-bearing mice treated with CHMACS was significantly higher, while that of *uncultured_bacterium_g_Coriobacteriaceae_UCG-002*, *uncultured_bacterium_g_Dubosiella*, *uncultured_bacterium_g_Lachnospiraceae_NK4A136_group*, and *anduncultured_bacterium_f_Lachnospiraceae* was significantly lower. Furthermore, cluster analyses indicated that in the CHMACS-treated group, *Verrucomicrobia*, *Spirochaetes*, *Fusobacteria*, *Patescibacteria*, and *Firmicutes* contributed the most to fecal microbial diversity. These results, particularly those related to *Fusobacteria* and *Patescibacteria*, were consistent with those of a previous study (Jia et al., 2021). Collectively, these data highlighted that CHMACS indeed plays a pharmacological role by modulating specific CC-associated flora.

Finally, as CHMACS was found to alter the distribution and structure of gut microbiota, we investigated its effects on the immune system. Recurrent colitis can reportedly lead to the development of CC. Colitis is usually caused by bacterial or viral infections, which activate the immune response, leading to initial inflammation and recurrent ulcers, followed by an atypical increase in aneuploid cells and epithelialization. In the intestinal tract, tumor-associated bone marrow cells secrete IL-23, which affects the polarization of Th17 cells, followed by the production of IL-17A, IL-21, TNF-α, and IL-6, which induce a tumor-promoting inflammatory response and promote CC development (Jia et al., 2021). Peuker et al. (2022) recently reported that microbiota-dependent activation of the myeloid calcineurin–NFAT pathway inhibited B7H3- and B7H4-dependent antitumor immunity in colorectal cancer in mice; moreover, recruitment of IFNγ-producing CD8^+^ T-cells to colonic mucosa relied on the presence of immunogenic bacteria in colonic ileal epithelial cells, as well as on the upregulation of specific chemokines (e.g., Cxcl10) and other IFN-induced genes in these cells. They also found that microbial sensing by myeloid cells promoted calcineurin- and NFAT-dependent IL-6 release, expression of the co-suppressor molecules B7H3 and B7H4 in tumor cells, and inhibition of CD8^+^ T-cell-dependent antitumor immunity (Peuker et al., 2022). After a comprehensive evaluation of changes caused upon CHMACS treatment, and based on the results of computational analyses, we speculate that CHMACS promotes amino acid metabolism and suppresses carbohydrate metabolism. Metabolite-related changes seem to be closely related to the maturation and activation of immune cells (Figure 9C).

To conclude, we believe that CHMACS activates immune cells, which consequently kill CC cells, by regulating the gut microbiota–Th17 axis.

## Data Availability

The raw data supporting the conclusion of this article will be made available by the authors, without undue reservation.
